# Reciprocal association between neurovascular conflict and trigeminal neuralgia: a systematic review and meta-analysis

**DOI:** 10.22514/jofph.2026.035

**Published:** 2026-05-12

**Authors:** Raimundas Golubevas, Augustinas Buškus, Gintaras Janužis, Dainius Razukevičius, Jan Pavel Rokicki

**Affiliations:** ^1^Department of Maxillofacial Surgery, Medical Academy, Lithuanian University of Health Sciences, LT-54308 Kaunas, Lithuania

**Keywords:** Trigeminal neuralgia, Neurovascular conflict, Trigeminal nerve, Magnetic resonance imaging

## Abstract

**Background**: Classical trigeminal neuralgia is thought to be primarily caused by neurovascular contact of the trigeminal nerve root. However, neurovascular contact is also seen in individuals without trigeminal neuralgia on magnetic resonance imaging (MRI). Understanding this reciprocal association is important for clinical decision-making, as microvascular decompression is usually considered in medication-refractory cases. **Methods**: We performed a systematic review and meta-analysis of MRI studies evaluating neurovascular contact in patients with trigeminal neuralgia and in individuals without trigeminal neuralgia. PubMed, ScienceDirect, and the Cochrane Library were searched for studies published between 2015 and 2025. Seven studies involving 699 patients and 1092 trigeminal nerves (357 symptomatic and 735 asymptomatic) met the inclusion criteria and were analyzed. **Results**: Neurovascular contact was present in 66.7% of nerves in individuals without trigeminal neuralgia (95% confidence interval: 58.9–73.8%) and in 87.5% of nerves in patients with trigeminal neuralgia (95% CI: 81.3–91.8%). The symptomatic side showed neurovascular contact at significantly higher rates than asymptomatic nerves. Severe contact was rarely observed in non-trigeminal neuralgia patients, but was strongly associated with symptomatic nerves. Neurovascular contact at the root entry zone was not significantly linked to the symptomatic side. Interpretation across studies was limited by heterogeneous and non-standardized reporting of neurovascular contact location and severity. **Conclusions**: Simple neurovascular contact is common in individuals without trigeminal neuralgia, whereas severe contact is more specific to symptomatic nerves, supporting a reciprocal association between neurovascular conflict and trigeminal neuralgia. Neurovascular contact should not be regarded as a binary MRI finding; severity, location, and vessel type appear to be important for symptom development. Standardized MRI reporting protocols and larger, well-designed studies are needed to refine diagnostic criteria and imaging-based assessment of trigeminal neuralgia. **The PROSPERO Registration**: CRD420250611313.

## 1. Introduction

The 3rd edition of the International Classification of Headache Disorders (ICHD) describes trigeminal neuralgia (TNN) as recurrent unilateral brief electric shock-like pains, abrupt in onset and termination, limited to the distribution of one or more divisions of the trigeminal nerve (TN) [1]. The condition is severely disabling for patients, disturbing their everyday activities and considerably harming their psychosocial wellbeing [2]. Epidemiological studies report incidence rates of 5.9 per 100,000 person-years (PY) in females, with the rate being slightly lower in males (3.4 per 100,000 PY), and with the incidence increasing with age [3].

TNN, per ICHD, is classified into classical, or primary, secondary, and idiopathic [1]. Secondary TNN may be caused by an underlying pathology or abnormality via structural compression; moreover, viral, fungal, and bacterial infections and inflammatory disorders may also trigger this condition [1, 4, 5]. Classical TNN is thought to be primarily caused by neurovascular conflict (NVC) of the TN root [6]. NVC is described as immediate contact between a nerve and a blood vessel with ensuing compression, displacement, and/or atrophy of the affected nerve [7]. The inflicted morphological changes on the TN by a blood vessel, typically an artery, are thought to produce local microstructural abnormalities—focal demyelination, which renders TN axons in close apposition without an insulative layer [8, 9]. This may produce ephaptic transmission of nerve impulses with aberrant impulse generation, triggering the painful condition [10]. The demyelination process is thought to be triggered by either the direct degenerative effect of astrocytes and oligodendrocytes around the pulsatile compression, or it may be secondary to endoneurial degenerative changes induced by the compressive ischemia [9].

High-resolution magnetic resonance imaging (MRI) techniques—particularly constructive interference in steady state (CISS) and other balanced steady-state free precession sequences such as true fast imaging with steady state precession (trueFISP) or fast imaging employing steady-state acquisition (FIESTA)—together with time-of-flight magnetic resonance angiography, allow excellent visualization of the trigeminal nerve and adjacent vascular structures and are routinely used to assess neurovascular contact [11, 12, 13].

Evaluation of NVC in TNN patients is crucial for appropriate clinical management, as the neurosurgical procedure of microvascular decompression (MVD) is generally considered in medication refractory cases [14]. Although the occurrence of NVC is seen in a large proportion of TNN patients (>57%), individuals without TNN, during MRI examinations of TN cranial portion due to other conditions (vertigo, sensorineural hearing loss, hemifacial spasms, *etc.*) are also reported to show NVC of the TN at substantial rates [15]. It has been previously reported that the NVC of TN in asymptomatic nerves of TNN individuals is severe much less frequently than in symptomatic nerves [7].

As previous studies did not account for studies that cover NVC in non-TNN patient cohorts, we aimed to assess both TNN and non-TNN individuals [7]. In this systematic review and meta-analysis, we explore the bidirectionality of TNN and NVC by exploring NVC in terms of its presence, severity, type, and location.

## 2. Materials and methods

### 2.1 Protocol and registration

This systematic review and meta-analysis aimed to evaluate the association between neurovascular conflict and the occurrence of TNN. The protocol for this study was documented prior to the initiation of the review in the The International Prospective Register of Systematic Reviews (PROSPERO), ID CRD420250611313. The Preferred Reporting Items for Systematic Reviews and Meta-Analyses (PRISMA) Statement was followed in reporting our systematic review and meta-analysis (**Supplementary material 1**) [16]. An electronic database search was conducted, including PubMed, ScienceDirect, and The Cochrane Library. The review of studies took place from 11 March to 25 March 2025. The search query used to gather relevant resources included the terms: magnetic resonance imaging, neurovascular compression, trigeminal neuralgia. Search strings in full and excluded articles are provided in **Supplementary Tables 1,2**. As full search strings were not available in full during editorial process, they were reconstructed based on the draft of the article.

### 2.2 Focus question

The focus question of this review was developed according to PICOS study design method: population (P), intervention (I), comparison (C) and outcome (O)—How neurovascular compression is distributed among patients, diagnosed with trigeminal neuralgia and trigeminal neuralgia-free patients? Table 1 demonstrates focus question development according to the PICOS study design.

**Table 1.** PICOS study design.

PICOS Criteria	Description
P (Population)	Patients with diagnosed trigeminal neuralgia
I (Intervention)	Presence of neurovascular contact of the trigeminal nerve observed in Magnetic Resonance Imaging
C (Comparison)	Patients presenting neurovascular conflict of the trigeminal nerve in Magnetic Resonance Imaging without symptoms of trigeminal neuralgia or facial pain
O (Outcome)	Trigeminal neuralgia expression
PICO	How is neurovascular compression distributed among trigeminal neuralgia patients?

### 2.3 Study selection

Two independent researchers conducted the evaluation of the search results. All articles were screened and excluded after investigating titles and abstracts and by means of filter application. Only studies published in the last 10 years (from 01 January 2015, up to 28 February 2025) were considered. Letters, animal studies, *in vitro* studies, case reports, editorials, systematic reviews and meta-analyses were excluded. Articles that were considered for final stage analysis were evaluated by full-text analysis and careful reviewing according to the eligibility criteria: *in vivo* studies published in the English language, evaluating the presence of neurovascular contact in MRI studies in patients complaining of TNN and asymptomatic individuals, studies comparing symptomatic and asymptomatic sides of TNN patients, and studies investigating the occurrence of TN vascular compression in patients without prior history of TNN. Studies that evaluated MRI results of patients who were to undergo microvascular decompression because of TNN were excluded due to selection bias of patients’ base. No appropriate institutional committee approval was required for this study according to local guidelines. Extracted numerical data is available in the Tables provided in the article.

### 2.4 Assessment of methodological quality

After careful systematic selection of studies, study protocol’s quality was assessed by full-text analysis, applying Joanna Briggs Institute (JBI) Critical Appraisal Checklist for risk of bias evaluation according to appropriate questionnaire type based on study type [17].

### 2.5 Statistical analysis

Meta-analysis was conducted using R (version 4.4.2) and RStudio (2024.12.0 Build 467) with the meta package (version 8.0.2) [18]. Proportions were analyzed using the metaprop function with the logit transformation (PLOGIT) for the following: (1) NVC occurrence in non-TNN and TNN patients; (2) NVC occurrence in non-TNN nerves. Comparisons for subgroup binary outcomes were made using the metabin function for the following data: (1) NVC occurrence in symptomatic *vs.* asymptomatic nerves in TNN individuals; (2) Severe *vs.* simple NVC occurrence in non-TNN individuals; (3) Arterial *vs.* venous involvement in symptomatic *vs.* asymptomatic nerves of TNN patients; (4) NVC occurrence in the root entry zone (REZ) *vs.* elsewhere in symptomatic *vs.* asymptomatic nerves of TNN individuals. We calculated the pooled odds ratios (ORs) with 95% confidence intervals (CIs). Heterogeneity was assessed using the *I*^2^ statistic and tau^2^, with the Mantel-Haenszel (MH) method applied for fixed-effect models in cases of low heterogeneity (*I*^2^ < 25%) and the Der Simonian-Laird (DL) method for random-effects models in cases of high heterogeneity (*I*^2^ ≥ 25%). Several sensitivity checks were attempted by excluding individual studies; however, removing studies substantially increased heterogeneity and did not improve the stability of the pooled estimates.

Quantitative assessment of publication bias was performed using Egger’s linear regression test (metabias function). Funnel plots were generated for the symptomatic and asymptomatic nerves. Because each subgroup contained fewer than ten studies, an adjusted minimum study threshold (min = 3) was used, and results were interpreted with caution.

## 3. Results

### 3.1 Study selection

Primary database search yielded 1098 studies. Removal of duplicates resulted in 1013 studies. After a thorough title and abstract screening, 20 were considered for full text analysis. However, 13 studies were rejected, with reasons stated in flow diagram, Fig. 1. The final search process returned 7 relevant studies which were included in qualitative and quantitative data synthesis. PRISMA flow diagram is demonstrated in Fig. 1.

**Fig. 1.** PRISMA flow diagram representing the study selection process. TN: trigeminal nerve; TNN: trigeminal neuralgia; MVD: microvascular decompression.

### 3.2 Study characteristics

7 studies were included in the qualitative synthesis. In sum, the included studies involved 699 patients, ranging from 27 to 141 patients observed per study. The MRI sequences were examined either by neuroradiologist or a neurosurgeon. 5 studies utilized 3-T MRI [19, 20, 21, 22, 23], and the remaining two studies relied on 1.5-T MRI results [12, 24]. Analyzed studies can be grouped into the following categories: studies that observed solely TNN patients, studies that observed both TNN patients and non-TNN patients, and studies that observed only non-TNN patients. A total of 357 symptomatic nerves and a total of 735 asymptomatic nerves were examined. Across the included studies, 51 of 357 symptomatic nerves and 165 of 735 asymptomatic nerves were examined using 1.5-T MRI, with the remaining nerves assessed using 3-T MRI systems. The asymptomatic nerves included 432 nerves from non-TNN patients and 303 nerves of asymptomatic side of TNN patients. Some studies differentiated the presence of NVC in symptomatic and asymptomatic sides of symptomatic patients [19, 20, 23]. A few studies assessed solely MRIs of patients without TNN or facial pain history [21, 22, 24]. Other studies relied on results from solely TNN sufferers [19, 23]. Others were case controlled studies with individuals without facial pain symptoms as controls [12, 20].

Studies differed in methodology of describing observed endpoints, and additional data regarding the presence and absence of NVC. Most studies described the presence and absence of NVC in a way that allowed for bilateral differentiation of the two sides of the cranial part of TN [19, 20, 21, 23]. The remaining studies described the occurrence of NVC in a way which does not permit differentiation between the hemispheric sides [12, 22, 24]. High heterogeneity was seen in how the severity of NVC is described and a variation of terms were utilized to describe the same condition. For the purposes of this review, we followed the operational definition used in the imaging-based study by Maarbjerg *et al*. [19], in which the REZ was defined as the proximal 7 mm of the trigeminal nerve from its entry into the pons. This definition is widely applied in neuroimaging studies of TN. It does not correspond exactly to the anatomical transition zone as described in cadaveric studies where the central–peripheral myelin junction is located within the first 1–4 mm of the cisternal segment [25]. A thorough assessment of the main characteristics, differences in methodology, and description of observed endpoints of studies is provided in **Supplementary Table 3** (Ref. [12, 19, 20, 21, 22, 23, 24, 26, 27]).

### 3.3 Qualitative and quantitative assessment of included studies

For risk of bias assessment, we utilized The Joanna Briggs Institute (JBI) Critical Appraisal Checklist for Analytical Cross-Sectional Studies and Case-Controlled studies, based on the study type [17]. The analyzed studies had low to medium levels of bias. Medium risk of bias was observed due to the unclearly identified or not stated confounding factors [12, 20, 22]. A thorough assessment of the risk of bias is provided in Table 2 (Ref. [12, 19, 20, 21, 22, 23, 24]).

**Table 2.** Risk assessment of the included studies based on the Joanna Briggs institute critical appraisal checklist for analytical cross-sectional studies and case-controlled studies.

Study/Question	JBI assessment tool	Q1	Q2	Q3	Q4	Q5	Q6	Q7	Q8	Q9	Q10
V. Maurya *et al*. [12], 2019	Case-controlled	Y	Y	Y	Y	Y	?	?	Y	Y	Y
S. Maarbjerg *et al*. [19], 2015	Cross-sectional	Y	Y	Y	Y	Y	Y	Y	Y	-	-
J. Docampo *et al*. [20], 2015	Case-Controlled	Y	Y	Y	Y	Y	?	?	Y	Y	Y
F. Ruiz-Juretschke *et al*. [21], 2019	Cross-sectional	Y	Y	Y	Y	Y	Y	Y	Y	-	-
R. Jani *et al*. [22], 2019	Cross-Sectional	Y	Y	Y	Y	Y	?	Y	Y	-	-
Kumar *et al*. [24], 2025	Cross-sectional	Y	Y	Y	Y	Y	Y	Y	Y	-	-
Li *et al*. [23], 2024	Cross-sectional	Y	Y	Y	Y	Y	Y	Y	Y	-	-

*Q1–Q10: Questions 1 to 10. -: not applicable; Y: Yes; ?: Unclear; JBI: Joanna Briggs Institute.*

Funnel plots for the non-TN and TN subgroups (**Supplementary Figs. 1,2**) did not demonstrate marked asymmetry. Egger’s linear regression test showed no evidence of small-study effects in either subgroup (non-TN: *t* = −0.36, *p* = 0.778; TN: *t* = −0.89, *p* = 0.466). Begg’s rank correlation test was likewise non-significant (non-TN: *z* = −0.52, *p* = 0.602; TN: *z* = −0.68, *p* = 0.497). These findings indicate no detectable publication bias, although the small number of available studies limits the sensitivity of asymmetry detection.

### 3.4 Evaluation of presence and absence, severity and associated vessel of NVC

7 publications that met the inclusion criteria were [12, 19, 20, 21, 22, 23, 24]. According to the results, we systematized the analysis process into separate criteria.

### 3.5 Occurrence rates of NVC

Case-controlled studies comparing symptomatic and asymptomatic patients show similar results, highlighting the high prevalence of reported NVC: Maurya *et al*. [12] report 80.4% occurrence of NVC in TNN patients while only 28.3% controls show NVC (χ^2^ = 29.945; *p* < 0.001), while Docampo *et al*. [20] report 83.3% NVC presence in symptomatic patients and 36% in controls. Contrasted to symptomatic versus asymptomatic sides of the same individual TNN patients, results of prevalence of any severity NVC present vary more widely among studies: 89% *vs.* 78% (symptomatic *vs.* asymptomatic side) (*p* < 0.05) [19], 83% *vs.* 40% [20], 92.2% *vs.* 75.4% [23]. Maarbjerg *et al*. [19] showed a statistically significant correlation between NVC occurrence side with pain side (OR = 2.4 (95% CI 1.2–4.8, *p* < 0.05)).

A notably high occurrence of NVC is seen in non-TNN patients’ observational studies: in Ruiz-Juretschke *et al*. [21] study, 71% of TNs showed some degree of NVC and 75% of the examined subjects with NVC had bilateral presence. Similar findings were presented by Jani *et al*.’s [22] study; authors noted a 67% occurrence of NVC in non-TNN patients. Kumar *et al*. [24] reported a 61% occurrence rate of NVC. Data from studies is summarized in Table 3 (Ref. [12, 19, 20, 21, 22, 23, 24]).

**Table 3.** Data on the occurrence of neurovascular conflict in trigeminal nerves based on patients’ magnetic resonance imaging.

Publication	Total Subjects	Patients with diagnosed TNN	NVC in TNN patients’ symptomatic nerves	NVC in TNN patients’ asymptomatic nerves	TNN patients’ asymptomatic nerves observed	Patients without TNN	Asymptomatic patients’ asymptomatic nerves	NVC observed in asymptomatic patients’ asymptomatic nerves
V. Maurya *et al*. [12], 2019	111	51	41	-	-	60	Unclear	17
S. Maarbjerg *et al*. [19], 2015	135	135	120	105	135	-	-	-
J. Docampo *et al*. [20], 2015	80	30	25	12	30	50	100	36
F. Ruiz-Juretschke *et al*. [21], 2019	100	-	-	-	-	100	200	142
R. Jani *et al*. [22], 2019	27	-	-	-	-	27	27	18
Kumar *et al*. [24], 2025	105	-	-	-	-	105	105	64
Li *et al*. [23], 2024	141	141	130	104	138	-		-
SUM	699	357	316	221	303	342	432	277

*Data is provided in numbers of patients or nerves. TNN: Trigeminal neuralgia; NVC: Neurovascular conflict.*

### 3.6 Severity

A marked difference was seen in the amount of NVC cases presenting severe contact—distortion of TN was seen in 24% *vs.* 2% (χ^2^ = 12.74, *p* < 0.05) cases *vs.* asymptomatic controls, imprinting/grooving in 51% *vs.* 2% (χ^2^ = 36.42, *p* < 0.05), thinning of caliber in 53% *vs.* 2% (χ^2^ = 38.42, *p* < 0.05) as reported by Maurya *et al*. [12]. Similarly, Docampo *et al*. [20] observed imprinting more frequently in symptomatic patients, accounting for 44% of NVCs in the TNN group, and only 2.7% in asymptomatic controls. Maarbjerg and colleagues showed that severe NVC was seen in 59.1% of TNN patients, while only 17% of asymptomatic sides of TNN patients showed severe involvement [19]. Such results line in agreement with Docampo *et al*. [20] and Li *et al*. [23] studies, with the former reporting 8.3% cases showing imprinting NVC in asymptomatic side of TNN patients and the latter presenting a Level IV involvement in only 17% of asymptomatic sides.

Regarding studies that examined solely non-TNN patients, severe NVC is rarely observed. Ruiz-Juretschke *et al*. [21] report a 0.07% incidence rate of Grade III (according to Sindou *et al*. [26] 2007) NVC in patients with no history of facial pain. Jani and colleagues found that none of the 18 NVCs were deforming and 22.2% were compressing, while Kumar *et al*. [24] reported a compressing NVC in 3.1% cases and a displacing one in 12.5% of cases [22, 24]. Data collected from the studies is provided in Table 4 (Ref. [12, 19, 20, 21, 22, 23, 24, 26, 27]).

**Table 4.** Data on the severity of neurovascular conflict in trigeminal nerves based on patients’ magnetic resonance imaging.

	V. Maurya *et al*. [12], 2019	S. Maarbjerg *et al*. [19], 2015	J. Docampo *et al*. [20], 2015	F. Ruiz-Juretschke *et al*. [21], 2019	R. Jani *et al*. [22], 2019	Kumar *et al*. [24], 2025	Li *et al*. [23], 2024
Severity	Thinning in caliber/Imprinting/Distortion	Simple/Severe (atrophy or displacement)	Contact/Imprint	Grading based on Sindou *et al*. [26], 2007: Grade I–III	Contact/Compression/Deformity	Grading based on Sindou *et al*. [27], 2002: Abutting/Displacing/Compressing	Level I—no contact/Level II—contact/Level III—Compression/Level IV—displacement (atrophy, thinning)
Data, TNN subjects’ symptomatic nerves, n	27/26/12	49/71	14/11	-	-	-	23/55/52
Total TNN subjects’ symptomatic nerves observed, n	41	120	25	-	-	-	130
Data, TNN subjects’ asymptomatic nerves, n	-	87/18	11/1	-	-	-	37/50/17
Total TNN subjects’ asymptomatic nerves observed, n	-	105	12	-	-	-	104
Data, non-TNN subjects’ asymptomatic nerves, n	1/1/1	-	35/1	131/10/1	14/4/0	54/8/2	-
Total non-TNN subjects’ asymptomatic nerves observed, n	17	-	36	142	18	64	-

*Data is provided in numbers of patients or nerves. TNN: Trigeminal neuralgia.*

### 3.7 Associated vessel

Regarding the offending vessel seen in TNN patients’ symptomatic side, arterial contacts were observed most frequently, specifically, Superior Cerebellar Artery (SCA) contact. Maurya *et al*. [12] showed a 56% incidence of SCA contact in TNN patients, contrasting to 21% in non-TNN patients and a statistically significant association (χ^2^ = 12.95, *p* < 0.05). Similarly, Docampo *et al*. [20] showed that SCA was responsible for 48% of NVCs in symptomatic patients. Maarbjerg *et al*. [19] did not differentiate between vessels, but reported that in 56% of TNN patients, NVC involved an artery, while arterial contact was seen in 38% of asymptomatic sides of TNN sufferers with a significant association between the factors with *p* < 0.05. Venous contact was observed less frequently, seen in 15% of cases and 24% of cases of TNN NVC as shown by Maarbjerg *et al*. [19] and Docampo *et al*. [20] respectively.

Patients without facial pain history but with an NVC identified tended to show venous NVC more frequently. Ruiz-Juretschke *et al*. [21] identified venous NVC in 66.2% of cases while Kumar *et al*. [24] reported a vein being responsible for 92% of non-TNN patients NVC. Maurya *et al*. [12] study results fall out of line with the previous two authors’ studies, showing none of the non-TNN patients showing venous contacts and majority—85.7% being SCA related [12]. The collected data is presented in Table 5 (Ref. [12, 19, 20, 21, 22, 23, 24]).

**Table 5.** Data on the vessel associated with neurovascular conflict in trigeminal nerves based on patients’ magnetic resonance imaging.

	V. Maurya *et al*. [12], 2019	S. Maarbjerg *et al*. [19], 2015	J. Docampo *et al*. [20], 2015	F. Ruiz-Juretschke *et al*. [21], 2019	R. Jani *et al*. [22], 2019	Kumar *et al*. [24], 2025	Li *et al*. [23], 2024
NVC vessel identification	SCA, AICA, SCA and AICA	Arterial/Veinous/Mixed*	SCA/AICA/Vein/VA/PICA/Mixed*	Venous/SCA/AICA/Mixed*	-	Vein/SCA	-
Data, TNN subjects’ symptomatic nerves, n	SCA = 25, AICA = 7, SCA and AICA = 3	Arterial = 76, Venous = 20, Mixed* = 24	SCA = 12, AICA = 1, Vein = 6, VA = 1, PICA = 0, Mixed* = 5	-	-	-	-
Total TNN subjects’ symptomatic nerves observed, n	35	120	25	-	-	-	-
Data, TNN subjects’ asymptomatic nerves, n	-	Arterial = 52, Venous = 29, Mixed* = 24	SCA = 5, AICA = 0, Vein = 7, VA = 0, PICA = 0, Mixed* = 0	-	-	-	-
Total TNN subjects’ asymptomatic nerves observed, n	-	105	12	-	-	-	-
Data, non-TNN subjects’ asymptomatic nerves, n	SCA = 25, AICA = 7, SCA and AICA = 3	-	SCA = 14, AICA = 3, Vein = 19, VA = 0, PICA = 0, Mixed* = 5	Venous = 94, SCA = 40, AICA = 3, Mixed* = 5	-	Vein = 59, SCA = 5	-
Total non-TNN subjects’ asymptomatic nerves observed, n	14	-	36	142	-	64	-

*The NVC is associated with both an artery and a vein. TNN: Trigeminal neuralgia; NVC: Neurovascular conflict; SCA: Superior Cerebellar Artery; AICA: Anterior Inferior Cerebellar Artery; VA: Vertebral Artery; PICA: Posterior Inferior Cerebellar Artery.

### 3.8 NVC location appraisal

Studies assessing the location of neurovascular contact (NVC) within the REZ have reported inconsistent findings regarding its association with the symptomatic side in trigeminal neuralgia. Maurya *et al*. [12] reported the mean distance of NVC to be 3.16 mm from the entry to Pons with an standard deviation (SD) of 2.33. The result was statistically significant compared with 5.71 mm mean distance of non-TNN patients’ NVC location with *p* < 0.05 [12]. Maarbjerg *et al*. [19] reported REZ NVC in 90.8% of cases in symptomatic nerves and 90.5% in asymptomatic nerves of TNN patients with no statistically significant correlation. This contrasts with Li *et al*.’s [23] findings, where researchers presented 78.7% of NVCs occurring in Near segment of the symptomatic nerves and much less frequently—39.1% of cases—in asymptomatic nerves of TNN patients.

Research articles that examined non-TNN patients report NVC in REZ at rates of 31% and 31.7% [21, 24]. More often, cisternal segment portion distal to the REZ is identified, accounting for 78.1% and 52.4% of cases as shown by different studies [21, 24].

### 3.9 Meta analysis

We first assessed the prevalence and different characteristics of NVC in TNN patients and asymptomatic (non-TNN) subjects. The overall prevalence of NVC was estimated across seven studies (n = 689). The NVC proportion in non-TNN subjects in 3 pooled studies was 66.7% (95% CI: 58.9–73.8%) and 87.5% (95% CI: 81.3–91.8%) symptomatic TNN individuals. A highly significant difference between the two groups was seen (*Q* = 17.70, *p* < 0.0001). Although the overall heterogeneity was high (*I*^2^ = 87.2%, *p* < 0.0001), separate subgroups showed moderate heterogeneity (*I*^2^ = 36.6% for non-TNN, 47.7% for TNN). This suggests that the TNN *vs.* non-TNN distinction explains much of the variability (Fig. 2, Ref. [12, 19, 20, 21, 22, 23, 24]).

**Fig. 2.** Forest plot demonstrating the proportions of neurovascular conflict (NVC) in patients diagnosed with trigeminal neuralgia (TN) and patients without trigeminal neuralgia (non-TN). *Q* = 17.70 (*p* < 0.05); non-TN NVC prevalence 66.7% (95% CI: 58.9–73.8%); TN NVC prevalence 87.5% (95% CI: 81.3–91.8%); Overall heterogeneity *I*^2^ = 87.2% (*p* < 0.05). CI: confidence intervals.

Analysis of TNN patients revealed, that NVC is significantly more frequently seen in symptomatic nerves than in asymptomatic nerves of same individuals (OR = 3.49, 95% CI: 1.97–6.20; *Z* = 4.27, *p* < 0.05) with moderate heterogeneity between studies (*I*^2^ = 35.5%, *p* = 0.2121) (Fig. 3, Ref. [19, 20, 23]). The pooling of 4 studies revealed that the total proportion of NVC occurrence among non-TNN individuals and TNN individuals’ asymptomatic nerves is 74.0% (95% CI: 70.0–77.0%) and is statistically significant (*Z* = 10.00, *p* < 0.05) with studies showing no heterogeneity (*I*^2^ = 0%, *p* = 0.4262) (Fig. 4, Ref. [19, 21, 22, 23]). This suggests a consistent baseline NVC prevalence in asymptomatic nerves across both populations, though lower than in symptomatic TNN nerves (87.5%).

**Fig. 3.** Forest plot demonstrating the Odds Ratio of Neurovascular conflict (NVC) in symptomatic *vs.* asymptomatic nerves of trigeminal neuralgia (TNN) patients. OR = 3.49 (95% CI: 1.97–6.20), *Z* = 4.27 (*p* < 0.05), *I*^2^ = 35.5% (*p* = 0.2121). OR: odds ratio; CI: confidence intervals.

**Fig. 4.** Forest plot demonstrating the prevalence of neurovascular conflict (NVC) in patients without trigeminal neuralgia (non-TNN). Proportion 74.0% (95% CI: 70.0–77.0%), *Z* = 10.00 (*p* < 0.05), *I*^2^ = 0% (*p* = 0.4262). CI: confidence intervals.

Non-TNN individuals with NVC detected tended to show severe contact significantly less frequently than non-severe contact (OR = 0.02, 95% CI: 0.01–0.10; *Z* = −5.26, *p* < 0.05), although substantial heterogeneity (*I*^2^ = 79.2%, *p* < 0.05) suggests potential influence from study design differences (Fig. 5, Ref. [21, 22, 24]). Arterial NVC was shown to be significantly more common than venous in TNN patients symptomatic when comparing with asymptomatic nerves (OR = 2.18, 95% CI: 1.21–3.94; *Z* = 2.60, *p* < 0.05) with low heterogeneity (*I*^2^ = 5.8%, *p* = 0.3029) (Fig. 6, Ref. [19, 20]). Regarding the location of detected NVC, an analysis of two studies presented no significant association between REZ NVC and NVC elsewhere when comparing symptomatic nerves versus asymptomatic nerves in TNN patients (OR = 0.73, 95% CI: 0.41–1.30; *Z* = −1.06, *p* = 0.2882) with studies showing no heterogeneity (*I*^2^ = 0.00%, *p* = 0.3179) (Fig. 7, Ref. [19, 23]).

**Fig. 5.** Forest plot demonstrating the Odds ratio analysis of severe versus non-severe neurovascular conflict occurrence in neuralgia-free patients. OR = 0.02 (95% CI: 0.01–0.10), *Z* = −5.26 (*p* < 0.05), *I*^2^ = 79.2% (*p* < 0.05). OR: odds ratio; CI: confidence intervals; NVC: Neurovascular conflict.

**Fig. 6.** Forest plot demonstrating the Odds ratio analysis of arterial versus venous neurovascular conflict in trigeminal neuralgia patients symptomatic and asymptomatic sides. OR = 2.18 (95% CI: 1.21–3.94), *Z* = 2.60 (*p* < 0.05), *I*^2^ = 5.8% (*p* = 0.3029). OR: odds ratio; CI: confidence intervals; TNN: trigeminal neuralgia.

**Fig. 7.** Forest plot demonstrating the Odds ratio analysis of neurovascular conflict (NVC) occurrence in symptomatic versus asymptomatic nerves of trigeminal neuralgia patients in root entry zone (REZ) versus elsewhere. OR = 0.73 (95% CI: 0.41–1.30), *Z* = −1.06 (*p* = 0.2882), *I*^2^ = 0.00% (*p* = 0.3179). OR: odds ratio; CI: confidence intervals.

## 4. Discussion

This study examined the occurrence rates, severity, location, and involved vessels in patients suffering from TNN symptomatic and asymptomatic nerves and patients without history of facial-pain related pathologies. We conducted a thorough review of 7 publications. Included articles involved either observation of MRI results of TNN patients, patients that have had MRI because of other pathologies that do not show history or current symptoms of TNN (controls) or both. Analyzed articles focused on prevalence of NVC, blood vessels involved in NVC, severity of NVC, and its location.

As early as 1934, NVC had been suspected to be a causal factor of TNN [28]. Yet, not all patients presenting neurovascular compression present symptomatic occurrence of TNN and it is a frequent finding in many asymptomatic individuals’ MRIs that are examined for different pathologies (Sensorineural hearing loss, vertigo, *etc.*), therefore, the theory of NVC induced TNN has been challenged [15, 21].

Our systematic review and meta-analysis support the strong association between NVC and TNN, consistent with previous studies [7, 29]. Multiple studies report higher prevalence of NVC in TNN patients compared with controls [12, 20]. This is further supported by meta-analysis results, showing a statistically significant 3.49-fold increased odds (95% CI = 19.97–6.20) of NVC being observed on the symptomatic side of TNN patients when contrasting to asymptomatic side with moderate heterogeneity across 3 studies. We also found that 87.5% of symptomatic TNN nerves exhibited NVC, compared with 66.7% of asymptomatic individuals nerves with a statistically significant difference. While this difference suggests a positive association between NVC and TNN symptoms, it also highlights that NVC is common in asymptomatic individuals (67.7%), implying that NVC alone may not be sufficient to cause symptoms in all cases and there may be a baseline NVC occurrence in healthy patients that could be regarded as a normal finding in general population. The high consistency of NVC occurrence highlights the likely effect of it being as one of the causal factors for TNN, however, regarding only the mere presence of NVC as a sole culprit of the neuralgia would be misleading. These findings suggest that treating NVC as a binary measure can result in potential misdiagnosis which may potentially lead to overtreatment when considering neurosurgery [24]. The NVC without TNN is postulated to be determined by the lack of severity of compression [30].

Our review and meta-analysis, in line with previous studies, suggests that it is crucial to assess the associated data when it comes to analyzing the results of MRI of TNN patients and the presence of NVC [15]. One factor that tends to be distinguishably more associated with symptomatic TNN nerves and less seen in asymptomatic nerves is the severity of NVC [30, 31]. Our systematic review showed that severe involvement is more frequently seen in symptomatic nerves. Authors consistently reported a severe NVC involvement being much more commonly associated with symptomatic nerves of TNN patients [12, 19, 20, 23]. Meta-analysis showed that individuals without history of facial pain show severe contact at considerably low rates (OR = 0.02), although this data may be influenced by high inter-study heterogeneity (*I*^2^ = 79.2%, *p* < 0.05). We were unable to analyze the differences in severity between symptomatic and asymptomatic nerves due to the differences in reported severity types.

The included studies defined REZ inconsistently, limiting comparability between datasets. Although previous reports suggested that symptom-causing NVC occurs more frequently within the REZ [25, 32], our meta-analysis did not demonstrate a significant association between REZ location and symptom laterality (OR = 0.73, 95% CI: 0.41–1.30). While qualitative evidence indicates a potential REZ predilection, the currently available pooled data are insufficient to support a statistically robust effect.

Regarding vessel types, authors noted SCA to be the most commonly implicated vessel in TNN [12, 20]. Arterial compression’s association with occurrence in TNN is explained as constant arterial pulsing induced focal demyelination of TN, a pathogenic mechanism of ephaptic transmission, leading to altered pain mechanisms with possibly ensuing neuropathic pain [12, 33]. These results fall in line with previous findings, showing that SCA most commonly produces NVC in the cisternal segment literature [34, 35]. Our meta-analysis also showed that arterial NVC was significantly associated with NVC when compared with venous NVC, occurring 2.18 times more frequently than venous in symptomatic nerves (OR = 2.18). There was high heterogeneity between the reported vessel types and differentiation. There was no method of merging the offending vessel type and the severity of the compression or location, as results on a singular patient basis were not readily available. More comprehensive data that associates severity, vessel type, location, and the occurrence or absence of trigeminal neuralgia on a single-nerve basis would allow for more intricate analysis in attempts to create an algorithm to objectively assess neurovascular conflict as the etiological factor in individual cases.

This systematic review and meta-analysis has certain shortcomings and limitations. In the data analysis, factors such as patient gender, presence of hypertension, and age were not considered. All these factors are associated with a higher incidence of TN [36, 37, 38, 39]. During study selection, publications were excluded if their patient population consisted of individuals who were scheduled for or had undergone MVD neurosurgical procedures due to TN or medication-resistant TN. However, a recently conducted meta-analysis investigating TN patients and NVC did not exclude such studies [7]. In the authors’ opinion, including such data leads to a preferential assessment of patients already diagnosed with TN and NVC, as TNN patients without NVC detected on MRI would simply not be considered during data collection. Additionally, this study did not account for technical differences between MRI machines or variations in the MRI segmentation methods used.

A severely limiting factor in merging data in attempts to analyze NVC presence and associated parameters in TNN patients, and healthy patients alike, is the differences in the ways authors categorize data. We observed the most differences in the way the severity of NVC is reported. The many ways of defining contact severity make it extremely difficult to draw conclusions when attempting meta-analysis. Furthermore, definitions of the root entry zone varied across studies, with imaging-based studies commonly using a 0–7 mm proximal segment, while anatomical work defines the central–peripheral myelin transition zone as a shorter 1–4 mm region. This definitional heterogeneity limits the comparability of REZ-specific findings. We believe there should be a recommended standardized protocol established for the analysis of NVC location, severity, and offending vessels.

In conclusion, although NVC can occur in healthy, asymptomatic individuals and a baseline of occurrence in patients without trigeminal neuralgia is seen, it is more frequently observed in patients with TNN. NVC should not be viewed as a binary criterion, since factors such as the severity of NVC, location, and the type of offending vessel are significant in the manifestation of TNN. Albeit at lower, nevertheless, significant rates, NVC does occur in asymptomatic individuals. Current evidence is insufficient to fully explain why some cases of NVC do not result in TN symptoms. Establishment of standardized protocol for reporting data on NVC discovered in MRI studies in TNN patients should be developed and large sample-size trials should be conducted to allow for more concrete conclusions to be stated and a standardized protocol for NVC assessment to be drawn.

## Supplementary Material

Supplementary material associated with this article can be found, in the online version, at https://files.jofph.com/ files/article/2054073858745352192/attachment/ Supplementary%20material.zip.




