# Supplementary material for: Reciprocal association between neurovascular conflict and trigeminal neuralgia: a systematic review and meta-analysis
Source: J Oral Facial Pain Headache. 2026 May 12;40(3):52–64. doi: 10.22514/jofph.2026.035 (PMC13223915; doi:10.22514/jofph.2026.035)
Supplement: Supplementary file 2 [file Supplementary-material-2.docx]

Supplementary material 2

Supplementary Table 1. Search strings for database search.

| Database | Exact search string | Additional filters |
| --- | --- | --- |
| PubMed | (“trigeminal neuralgia”[Mesh] OR “trigeminal neuralgia”[tiab] OR “trigeminal nerve”[tiab] OR “cranial nerve V”[tiab] OR neuralgia[tiab] OR “facial pain”[tiab] ) AND ( neurovascular[tiab] OR “neurovascular compression”[tiab] OR “neurovascular contact”[tiab] OR “vascular compression”[tiab] OR “vascular loop”[tiab] OR compression[tiab] OR contact[tiab] OR conflict[tiab]) AND ( MRI[tiab] OR “magnetic resonance”[tiab] OR “magnetic resonance imaging”[Mesh] OR imaging[tiab] OR “3 T”[tiab] OR “3T”[tiab] OR “3D”[tiab] OR “three-dimensional”[tiab] OR CISS[tiab] OR FIESTA[tiab]) | Publication Year (“2015/01/01”[dp]: “2025/02/28”[dp]); |
| Science Direct | “trigeminal neuralgia”AND (neurovascular OR compression)AND (MRI OR “magnetic resonance”) | Publication Year 2015–2025; Research Articles |
| Cochrane Library | matching (trigeminal neuralgia OR trigeminal nerve OR neuralgia) AND (neurovascular OR compression OR contact OR conflict OR “vascular loop”) AND (MRI OR “magnetic resonance” OR imaging) in Title Abstract Keyword | Year 2015–2025 |

Supplementary Table 2. Reasons for exclusion and excluded studies.

| Reason of Exclusion | Studies | Number of studies excluded |
| --- | --- | --- |
| Irrelevant Parameters of Trigeminal Nerve studied | MRI trigeminal nerve digitalization for trigeminal neuralgia diagnosis, Zheng *et al.* 2024 ([https://doi.org/10.1016/j.jrras.2024.101053](https://www.sciencedirect.com/science/article/pii/S1687850724002371?via%3Dihub));  MRI Findings in Trigeminal Neuralgia without Neurovascular Compression: Implications of Petrous Ridge and Trigeminal Nerve Angles, Zhong *et al.* 2022 ([https://doi.org/10.3348/kjr.2021.0771](https://kjronline.org/DOIx.php?id=10.3348/kjr.2021.0771)) | 2 |
| Patients with TNN with MVD history | The Role of Preoperative Magnetic Resonance Imaging in Assessing Neurovascular Compression Before Microvascular Decompression in Trigeminal Neuralgia, Xu *et al.*, 2022 ([https://doi.org/10.1016/j.wneu.2022.09.092](https://www.sciencedirect.com/science/article/abs/pii/S1878875022013699?via%3Dihub));  Evaluation of the correlation between trigeminal nerve atrophy and trigeminal neuralgia using multimodal image fusion: A single-center retrospective study, Li *et al.*, 2024 ([https://doi.org/10.1016/j.clineuro.2024.108387](https://www.sciencedirect.com/science/article/abs/pii/S0303846724002749?via%3Dihub));  Microvascular decompression for trigeminal neuralgia: A retrospective analysis of long-term outcomes and prognostic factors, Pascasio *et al.*, 2020 ([https://doi.org/10.1016/j.nrleng.2021.03.010](https://www.sciencedirect.com/science/article/pii/S2173580822001675?via%3Dihub));  The Use of MRI in Preoperative Decision-Making for Trigeminal Neuralgia: A Single-Center Study, Mooney *et al.*, 2021 ([https://doi.org/10.1016/j.wneu.2020.10.146](https://www.sciencedirect.com/science/article/abs/pii/S1878875020323482?via%3Dihub));  Magnetic resonance imaging evaluation of masticatory muscle changes in patients with primary trigeminal neuralgia before microvascular decompression, Zhang *et al.*, 2022 ([https://doi.org/10.1097/md.0000000000031010](https://journals.lww.com/md-journal/fulltext/2022/10140/magnetic_resonance_imaging_evaluation_of.122.aspx));  Microvascular decompression and MRI findings in trigeminal neuralgia and hemifacial spasm: single-center experience, Hitchon *et al.*, 2015 ([https://doi.org/10.1016/j.clineuro.2015.10.012](https://www.sciencedirect.com/science/article/abs/pii/S0303846715300433?via%3Dihub));  MRI of the Trigeminal Nerve in Patients With Trigeminal Neuralgia Secondary to Vascular Compression, Hughes *et al.*, 2016 ([https://doi.org/10.2214/ajr.14.14156](https://www.ajronline.org/doi/10.2214/AJR.14.14156));  Diffusion tensor imaging of microstructural alterations in the trigeminal nerve due to neurovascular contact/compression, Chai *et al.*, 2019 ([https://doi.org/10.1007/s00701-019-03851-2](https://link.springer.com/article/10.1007/s00701-019-03851-2));  Diffusion Tensor Imaging of Axonal and Myelin Changes in Classical Trigeminal Neuralgia, Zhang *et al.*, 2018 ([https://doi.org/10.1016/j.wneu.2018.01.095](https://www.sciencedirect.com/science/article/abs/pii/S1878875018301384?via%3Dihub)); | 1 |
| Drug effect on TNN studied | Predicting the therapeutic effect of carbamazepine in trigeminal neuralgia by analysis of neurovascular compression utilizing magnetic resonance cisternography, Tanaka *et al.*, 2019 ([https://doi.org/10.1016/j.ijom.2018.09.012](https://www.ijoms.com/article/S0901-5027(18)30382-5/abstract)) | 1 |
| Studies did not account for space-occupying lesions | Neuroanatomical Determinants of Secondary Trigeminal Neuralgia: Application of 7T Ultra-High Field Multimodal MRI, Arrighi-Allisan *et al.*, 2019 ([https://doi.org/10.1016/j.wneu.2019.11.130](https://www.sciencedirect.com/science/article/abs/pii/S1878875019329808?via%3Dihub)) | 1 |

Supplementary Table 3. End points description provided in the analyzed studies, methods, patient base, machinery and characteristics of studies.

|  | V. Maurya *et al.* [12], 2019 | S. Maarbjerg *et al.* [19], 2015 | J. Docampo *et al.* [20], 2015 | F. Ruiz-Juretschke *et al.* [21], 2018 | R. Jani *et al.* [22], 2018 | Kumar *et al.* [24], 2025 | Li *et al.* [23], 2024 |
| --- | --- | --- | --- | --- | --- | --- | --- |
| Type of study | Retrospective single-center case-controlled study | Prospective single-center observational study | Prospective single-center case-controlled study | Retrospective single-center observational study | Prospective single-center observational study | Retrospective single-center observational study | Retrospective single-center observational study |
| Patient groups | TNN patients (study) and SNHL patients (control) | classical TNN patients | TNN patients (study) and UHL (control) | non-TNN: hypoacusis, vertigo, dizziness or headache patients undergone MRI | non-TNN: patients to undergo MVD for hemifacial spasms | non-TNN: vertigo, dizziness, tinnitus or headache patients undergone MRI | TNN patients |
| MRI evaluating specialists | 2 neuroradiologists, blinded to symptomatic side | 1 neuroradiologist, blinded to symptomatic side | 2 neuroradiologists, blinded to symptomatic side | 1 neurosurgeon, 1 neuroradiologist | 1 neuroradiologist | 2 neuroradiologists | 2 neuroradiologists |
| MRI machinery* | 1.5T | 3T | 3T | 3T | 3T | 1.5T | 3T |
| MRI sequencing technique | 3D Constructive Interference in Steady-State | T2-Weighted Turbo-Spin-Echo; T2-Weighted Gradient and Spin-Echo; 3D Time-of-Flight Magnetic Resonance Angiography; 3D High-Resolution Heavily T2-Weighted Balanced Fast Field Echo | Fusion of 3D Fast Imaging Employing Steady-State Acquisition and 3D Time-of-Flight Magnetic Resonance Angiography | 3D Fast Imaging Employing Steady-State Acquisition | Steady-State Free Precession | 3D Fast Imaging Employing Steady-State Acquisition | T2-Weighted Mixed 3D Transverse Spectral Adiabatic Inversion Recovery |
| Described relevant end-points | NVC presence; NVC location; NVC severity; NVC vessel | NVC presence; NVC location; NVC severity; NVC vessel | NVC presence; NVC severity; NVC vessel | NVC presence; NVC location; NVC severity; NVC vessel | NVC presence; NVC location; NVC severity | NVC presence; NVC location; NVC severity; NVC vessel | NVC presence; NVC location; NVC severity |
| Unilateral or bilateral assessment of MRI results** | Unilateral | Bilateral | Bilateral | Bilateral | Unilateral | Unilateral | Bilateral |
| NVC severity appraisal*** | Thinning in caliber/Imprinting/Distortion | Simple/Severe (atrophy or displacement) | Contact/Imprint | Grading based on Sindou *et al.* [26], 2007: Grade I–III. | Contact/Compression/Deformity | Grading based on Sindou *et al.* [27], 2002: Abutting/Displacing/Compressing | Level I—no contact/Level II—contact/Level III—Compression/Level IV—displacement (atrophy, thinning) |
| NVC vessel identification^†^ | SCA, AICA, SCA and AICA | Arterial/Veinous/Mixed | SCA/AICA/Vein/VA/PICA/Mixed | Venous/SCA/AICA/Mixed | - | Vein/SCA | - |
| NVC location appraisal^‡^ | Distance from Pons, mm | REZ/Peripheral | - | REZ/Porous trigeminus/Cisternal Segment | - | REZ/Porous trigeminus/Cisternal Segment | Near segment/Far segment |

TNN: Trigeminal neuralgia; UHL: Unilateral hearing loss; MVD: Microvascular decompression; MRI: Magnetic Resonance Imaging; NVC: Neurovascular conflict; REZ: Root entry zone; SCA: Superior Cerebellar Artery; AICA: Anterior Inferior Cerebellar Artery; VA: Vertebral Artery; PICA: Posterior Inferior Cerebellar Artery. Notes: *Strength of magnetic field of the Magnetic Resonance Imaging machine utilized, either 1.5 Tesla of 3 Tesla; **Unilateral assessment—according to provided data from the study, a single side of the brain of each patient is described, Bilateral—the provided data in the study allows for bilateral differentiation of neurovascular conflict presence, absence and appropriate characteristics of each patient; ***Definitions and descriptions provided in the study utilized to grade the severity of neurovascular conflict of trigeminal nerve; ^†^Descriptions provided in the study utilized to identify the vessel present in neurovascular conflict; ^‡^Descriptions provided in the study utilized to identify the location of neurovascular conflict.


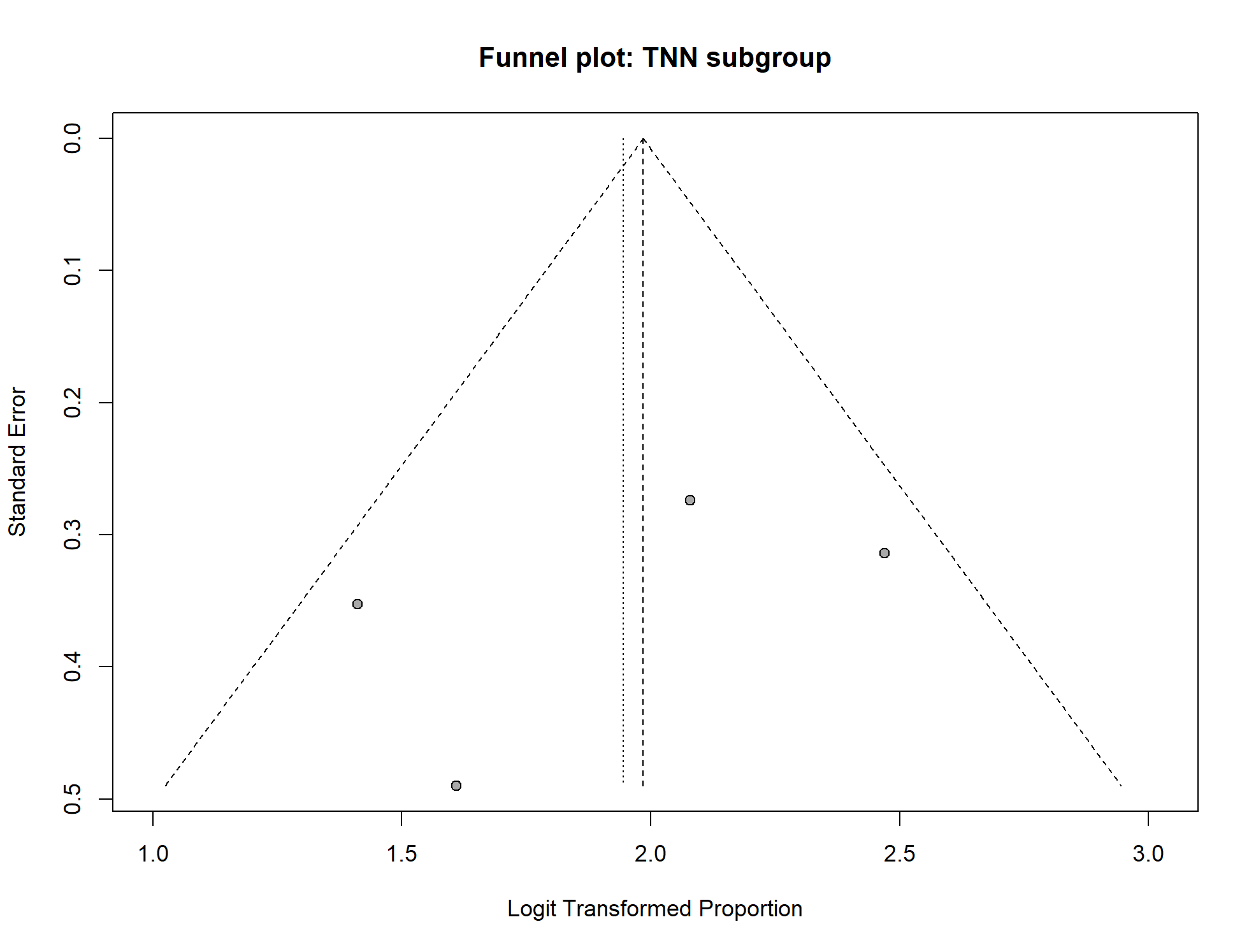


Supplementary Fig. 1. Funnel plot for TNN—Trigeminal Neuralgia nerves group *t* = −0.89, *p* = 0.466.


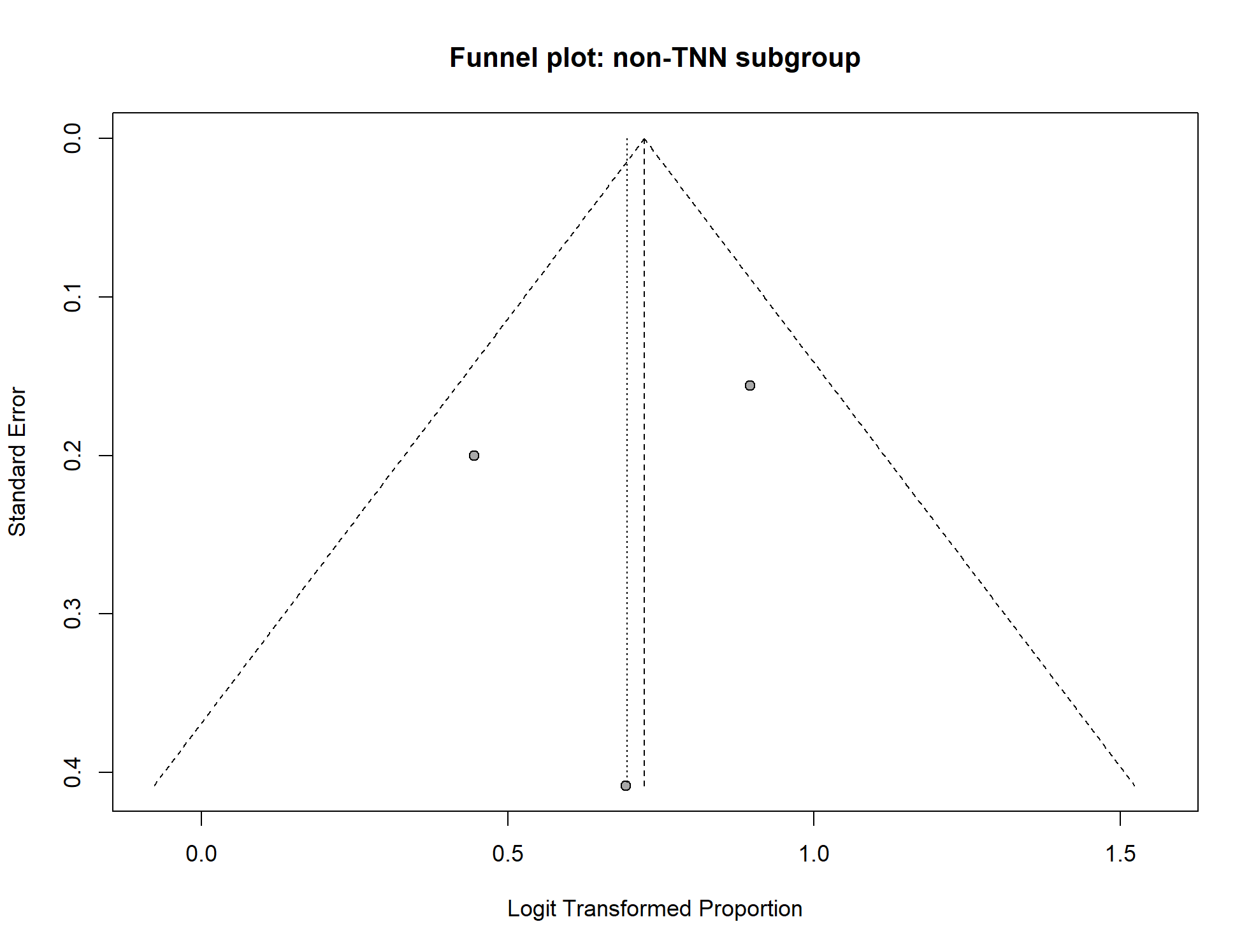


Supplementary Fig. 2. Funnel plot for non TNN—Trigeminal Neuralgia nerves group *t* = −0.36, *p* = 0.778.
